# Imagechain—Application of Blockchain Technology for Images

**DOI:** 10.3390/s21010082

**Published:** 2020-12-25

**Authors:** Katarzyna Koptyra, Marek R. Ogiela

**Affiliations:** Cryptography and Cognitive Informatics Laboratory, AGH University of Science and Technology, 30-059 Kraków, Poland; kkoptyra@agh.edu.pl

**Keywords:** imagechain, image watermarking, image cloud technologies

## Abstract

Imagechain is a cryptographic structure that chain digital images with hash links. The most important feature, which differentiates it from blockchain, is that the pictures are not stored inside the blocks. Instead, the block and the image are combined together in the embedding process. Therefore, the imagechain is built from standard graphic files that may be used in the same way as any other image, but additionally, each of them contains a data block that links it to a previous element of the chain. The presented solution does not require any additional files except the images themselves. It supports multiple file formats and embedding methods, which makes it portable and user-friendly. At the same time, the scheme provides a high level of security and resistance to forgery. This is achieved by hashing the whole file with embedded data, so the image cannot be altered or removed from the chain without losing integrity. This article describes the basic concept of an imagechain together with building blocks and applications. The two most important issues are embedding methods and block structure.

## 1. Introduction

Blockchain is a breakthrough technology used to create distributed databases. The data are stored in the form of a chain that grows incrementally, and the information, once saved, is immutable. Each block of data is connected to the previous one with a cryptographic hash function to ensure the integrity and prevent tampering with data (in fact, modifying even a single bit in the chain would require recomputing all hashes from the altered block to the last one, which is a power-consuming task). The main strength of blockchain lies in distribution because it makes the database directly accessible and resistant to DDoS attacks.

Blockchains usually store textual data. However, it is worth mentioning some efforts of chaining different data structures. The one idea was to place a digital image at the SteemIt [1], which normally only stores the texts in the blockchain. Instead of the default approach (uploading the image to the server), the author tried to embed base64-encoded binary image content into HTML. Unfortunately, this effort was unsuccessful due to technical limitations. There were also some projects focused on storing healthcare data. Authors of [2] proposed two blockchains—public and private—with different block structures. The system presented in [3] integrates blockchain with cloud infrastructure. Both papers made important contributions to the storage of index information and transaction records, but not the images themselves. Some other ideas of blockchain application are also worth mentioning. The aim of [4] was to store genomic data in blockchain to reward resource-sharing, remove the necessity of unnecessary mediators between the owners and users, promote collaborative work, and provide genome privacy. In [5], medical data in blockchain are shared between *n* servers and may be obtained when at least *t* participants collaborate. Additionally, the servers can perform homomorphic computations on the data. The authors of [6] propose a blockchain-based system of drug distribution to detect falsified and substandard drugs. Its main parts are distributed ledger, smart contracts repository and the history of documents and drug distribution. The application of blockchain in clinical trial data is discussed in [7]. It describes data management with smart contracts in the Ethereum network.

In blockchains, a common solution for protecting the integrity of a collection of files is the use of Merkle trees: the hashes of all files are organized in a tree, and the root of the tree is stored in the blockchain. It is efficient and suitable for large data structures. This concept was created by Ralph C. Merkle [8].

Blockchain technology also found application in IoT. It was involved in information management [9] to store data and access it with Etherum smart contracts. Another example was presented in [10] in which resources are controlled with digital currency, and the system rules are based on smart contracts. There are also projects aimed at limiting the required storage space by creating local blockchains and save constrained resources in IoT networks [11]. Other interesting works are focused on protocol optimization or adaptation to specific needs [12]. Unfortunately, not many papers consider digital pictures. We may found a blockchain-based medical image system [13] in which images are encrypted and may be retrieved with smart contracts. Some efforts have been made to place images in the interplanetary file system (IPFS) [14]—a distributed system for storing data in objects containing data and links. The problem with images is that they must be divided into parts of size up to 256 Kb, which is the limit set by a single IPFS object. A similar approach may be found in [15], where the authors presented an IoT-based solution for secure transmission and storage of digital images. Therefore, in existing solutions, the main trend is to divide the image and store its parts in the blockchain. This paper introduces imagechain—a new method of linking images. In contrast to other solutions, photos are not stored in a blockchain. Instead, they constitute the chain themselves. It is realized by saving the data directly in a graphical file. Therefore, the ledger is not a separated object but is rather embedded in the images.

The idea of an imagechain with an example of use is described in Section 2 and Section 3. The architecture of the system may be found in Section 4, which also contains details of block structure and a description of embedding techniques. Section 5 discusses security issues; Section 6 presents the analysis. Section 7 shows a few potential applications. Section 8 provides a comparison to other approaches and draws some conclusions. The last section summarizes the entire article.

## 2. An Idea of Imagechain

The motivation of this paper is to propose a new method of chaining digital images. The main assumption is that the solution should not require an external database. Therefore, this work is aimed to create a system in which images are linked directly to each other, and no other files are needed.

**Figure 1:**
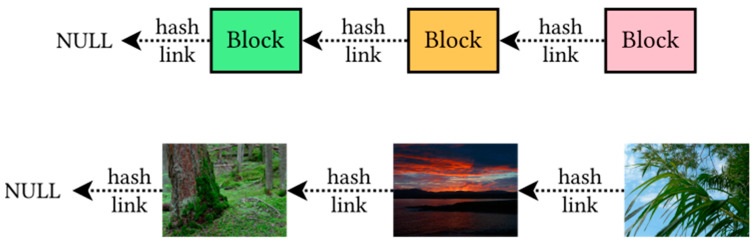
The comparison of the blockchain (**top**) and imagechain (**bottom**). In the imagechain, blocks are embedded in the images so that the pictures are directly linked to each other.

In general, the conception of an imagechain bears some similarity to the blockchain. As shown in Figure 1, they both form a linear structure connected with hash links. However, the imagechain no longer consists of text blocks, but instead, each chain element is an image linked to the previous one.

The links are made with hash values (fixed-length digests of arbitrary-size input). Each image contains an embedded piece of information that may be considered as a data block. These data block stores a hash of previous image file which links it to its predecessor. In some sense, imagechain reverses the blockchain paradigm: instead of storing images into chained blocks, chain information is embedded into images.

Two methods that are involved in imagechain architecture are embedding and extracting function. Their roles are complementary. The embedding function is used to put the block into the image. The extracting function, on the other hand, pulls the data out of the image. Both methods are publicly known to all participants as well as other system parameters (like a hash function).

## 3. Example of Imagechain

To demonstrate the described system in practice, a comprehensible example has been created. It uses simple blocks and familiar photos from Figure 1. The chosen hash function is SHA256. To start an imagechain, we select an initial image, which is the one entitled “climbing moss”. As it is located at the beginning of the chain, it does not have a predecessor, so its previous hash is equal to 0. Then we form the block containing all important pieces of information and embed it in the image (more technical details of this procedure are presented in the next section).

After this step, the chain has a length of 1. This value is then saved in the next photography as the previous hash. Such a process is repeated every time when subsequent images are appended to the chain. The elements are linked chronologically, and each of them has exactly one predecessor (except the first photo).

**Figure 2:**
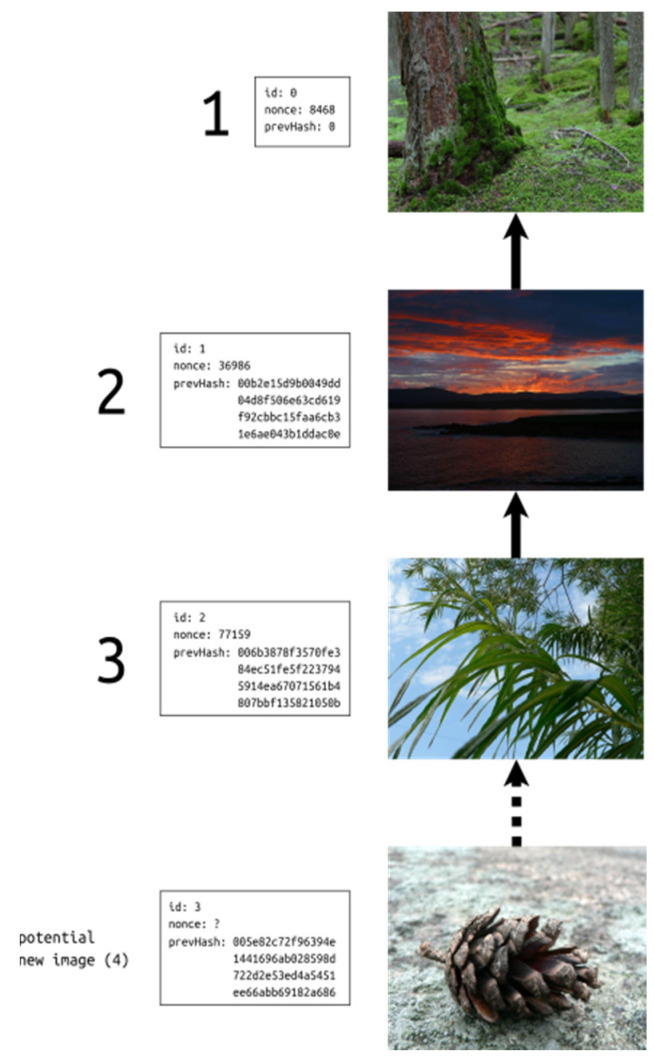
An example of a simple imagechain.

The result of chaining three images is shown in Figure 2. To extend this imagechain, one should compute the hash of the last element, create a new block and embed it in the incoming image. The new picture would become a new tail of the chain.

## 4. Creation of Data Blocks

Block structure and embedding function are important parts of imagechain architecture that should be taken into account when designing the system. They are discussed in this section.

### 4.1. Block Structure

A block is a piece of data in a structured format. Each block consists of (field, value) pairs, therefore the most natural and common choice is JSON, but it is technically possible to have blocks encoded in a different way.

The basic elements of each block is a hash value. It plays a crucial role in a chain link; in other words, it bonds the elements in a specific order. The block structure may be more complex, with a number of fields, depending on the application. The most common fields used for multiple purposes are as follows:

- Index is a numerical value indicating an order of the blocks. It is incremented by 1 every time when a new element is added to the chain;
- Timestamp denotes that some data existed on a particular date and time. This field is usually in the form of a number of seconds since 1 January 1970 00:00:00 UTC;
- The previous hash is the main and most important element of each block. It is used to verify if the content of the block has not been modified. Thus, the role of the hash value is not only to maintain the structure of the database but also to detect and prevent frauds. This is why the proper choice of the hash function is crucial in each chain system;
- Signature may be added to block when the system requires authorization or when the authorship is significant. In such cases, the algorithm is publicly known to participants. It is broadly used in cryptocurrencies;
- Nonce is a value that has no special meaning but may be modified to change the hash of the whole block. It is used in many public blockchains to control mining difficulty by imposing some requirements on the blocks (for example, only hashes with a fixed number of leading zeroes are considered as valid). Mining is the compute-intensive task of finding hashes meeting some additional conditions. It is used to validate the block in the chain;
- Additional data may contain a comment, description, etc. It depends purely on the application of the chain. The length of this field may be limited to prevent blocks from being too large.

The more information is present in the block; the more sophisticated functions may be implemented in the system. On the other hand, bigger blocks are disadvantageous as the size of the chain grows quickly, and storage problems arise. Therefore, a good practice is to design a system with minimal blocks tailored to the needs.

In the imagechain, the block size is also important because of the capacity of the carrier. The system architect should ensure that block size is adequate to be used with chosen algorithms. Section 4.2 and Section 6 give some insights into the recommended setup.

### 4.2. Embedding Method

**Figure 3:**
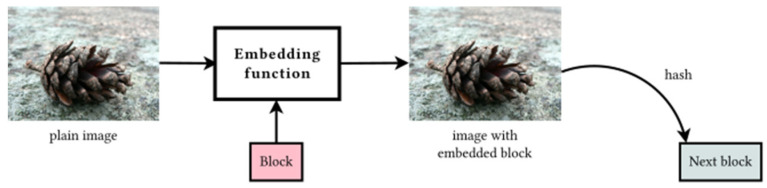
The role of the embedding function in the imagechain is to place data blocks into the picture. The hash of the output image is then included in the next block.

The proper functioning of the imagechain depends on embedding and extracting methods. These are complementary functions: the first is used for encoding the block into the image, and the second for retrieving it out. The embedding algorithm plays an important role in the whole system because the resulting image is hashed, and the digest becomes a part of a new block, which is later stored in another file, as presented in Figure 3.

The embedding functions may be similar to those used in watermarking or steganography [16,17]. However, in an imagechain, the requirements differ slightly from those two fields. Three features to consider are capacity, robustness and undetectability [18]. As they are competitive, each method focuses on excelling in one or being good in two of them. In watermarking, the most important thing is to make embedded data hard to remove. Additionally, the image should not be distorted, especially when the mark is invisible. Considering steganography, the detection of hidden secrets must be as difficult as possible. Here the container may be chosen freely, as it is not important per se. The capacity matters in both fields.

In imagechain, there are two main requirements crucial for all applications. First, as pictures are the main and essential part of the chain, they should not be distorted at all or, if necessary, modified in a minimal way. Second, the block must always fit into the image to provide a faultless operation of the system. Other characteristics, like robustness and undetectability, are, in most cases, useless and may be ignored because the chain should be immutable and all parameters of the system are overt to participants. The one exception from this is steganography, which is discussed in Section 7. In such a case, the embedding algorithm should assure high undetectability because the ledger’s existence is not revealed to the public.

**Figure 4:**
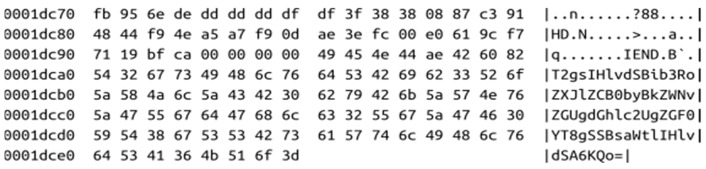
An example of data embedded in the PNG file after IEND marker (visible in hex dump).

The proper embedding methods for imagechain are container modification algorithms in which both the container (digital image) and the data (block) are given to a function that melts them together and returns a file with hidden data, exactly like previously drawn in Figure 2. This class of algorithms is divided into two subgroups: injection and substitution. In the former type of methods, the data are stored in normally unused parts of the file, for example, behind the EOF indicator. In the latter class, some parts of the container, like pixel bits or coefficients, are replaced with data to be hidden [19]. Substitution techniques have limited capacity and may cause very slight modifications of the picture, visible or not. On the other side, injection methods add extra data to the container and, as a result, the size of the file increases. However, the size of the image is, in most cases, significantly larger than the block, so this drawback would not present a serious problem. For this reason, the most suitable are injection algorithms, which are independent of the content, and maybe in some applications, the substitution methods can also be used. An example of the recommended approach is to store the data block in the JPEG comment section [20]. It may be later pulled out in the same way as all other photo metadata. Another good solution suitable for PNG (Portable Network Graphics) files is to place the data after the IEND marker (which marks the image end). These additional bytes are ignored when the file is opened in the image viewer but are visible in the hex editor, which is presented in Figure 4.

The embedding process is conducted every time when a new image is added to the chain. On the other hand, an extracting algorithm is used during verification or if someone wants to acquire additional data from the block. As verification is the most time-consuming part, the most essential to achieve high-performance and scalability is the proper selection of extracting (and complementary embedding) method. If the chain is expected to be long, fast injection algorithms are recommended.

It is possible to construct a chain that supports multiple image formats. In such a case, on the basis of the format, the program chooses the appropriate execution path and uses adequate embedding and extracting methods. Alternatively, the chain may consist of images in a single format; then, its implementation is straightforward. In both cases, images may be of different sizes, with one limitation. If the embedding algorithm does not offer unlimited capacity, the newly added image must have enough free space to store a data block.

As a general framework, imagechain does not force any specific consensus algorithm. Section 7 presents some possible applications which differ in terms of requirements for security, reliability, efficiency and scalability. We discuss some protocols from which one should select the most appropriate. Proof of work is one of the most popular consensus mechanisms used in Bitcoin and some other cryptocurrencies [21,22]. To add a new block, the users solve hard cryptographic challenges which are easy to verify once computed. Proof of work is sufficient for multiple applications of public imagechains. It provides high decentralization, makes attacks more expensive when the network grows and may be used anonymously. Unfortunately, mining is expensive and uses many resources. If this aspect matters in a specific application, other approaches are advised. In proof of stake, the chances of mining the next block depending on the amount of some good owned by users. This consensus mechanism was developed for cryptocurrencies when the stack is usually defined by the economic value of the coins [23]. Imagechain was not designed as a payment system, but in some applications, it may utilize proof of stake. When network participants may earn a reputation, the user with a high reputation has a larger fraction of the stack. This approach, however, may lead to an increase of centralization and discourage new users. In decentralized public imagechains, other solutions include proof of space or proof of retrievability, which rely on storage space [24,25]. When the network is not public, there are possible consensus algorithms based on trust [26]. Such systems have lower entry barriers and work well on a small scale but are vulnerable to the same problems as all trust-based systems [27,28]. The final decision on the consensus mechanism should take into consideration all strengths and threats in order to allow faultless operation of the network and protect it from adversaries.

## 5. Security Features

The issues faced by imagechain and blockchain are quite similar. The possible attacks on imagechain are forging an image or modify embedded block data. To conduct these attacks, the adversary needs to find a collision of the hash function. Therefore, collision resistance is the most important feature to consider during the design of the system. Another important aspect is the digest length. Some cryptographically secure hash functions produce long output, which may be a limitation if the embedding algorithm has a finite capacity. This is because digest length influences block size, and the block may be too big to fit into the container. Hence, the hash size should also be considered in security analysis. The hash function also plays a crucial role in verification. Therefore, a third essential aspect is its performance. The fast algorithms are recommended if scalability is demanded. However, the speed of hash functions may vary depending on hardware [1], so the final selection should take into account the intended platform.

In the imagechain, the whole carrier file is hashed, which means that both the picture and the block influence the resulting hash. This makes the imagechain very resistant to forgery. It follows that the same base image may be present in different chains, but, in the wake of block embedding, their digests would vary. Therefore, swapping visually identical pictures from different chains is trivial to detect.

It should be noted that in some imagechain applications, even tampered objects may still be detectable. Such situations occur when all images share common characteristics. For example, all of them depict buildings. With high probability, it may be supposed that a forged image would be very different from the remaining ones. If it contains random noise, the identification is possible with a visual inspection or entropy-based techniques.

Public imagechain may also be vulnerable to 51% attack and other problems similar to blockchain [3]. 51% attack is relatively difficult to conduct as it requires controlling the majority of the network. In theory, the attacker who gains so much control is able to tamper with data by dictating the consensus. Decentralized systems are defended by massive group mining efforts, which makes this attack time-consuming and expensive.

## 6. Analysis

The main feature of imagechain, which differentiates it from other approaches, is that digital images are linked directly with one another. There is no separated chain that stores only metadata or hyperlinks. Instead, the image itself becomes a new element of the chain. It is realized by embedding data block into the picture.

Comparing to other solutions, the imagechain shows some important advantages, which are as follows: The chained pictures are normal files, which may be used for different purposes, sent by email, uploaded on a website, etc. and still belong to the ledger. They do not need additional software to be viewed, so the whole solution is considered as user-friendly for non-technical users. On the other side, a program is required to conduct the verification process or to add a new image to the chain.

Another con of the proposed system is that it may be used in conspiracy. Some images added to a large database may be chained in a covert way known only to an initiated group of people. Such a situation requires the proper choice of embedding algorithm—its detection should be as hard as possible. Then it is possible to create a hidden imagechain that shows some relations between linked images that are not obvious to an external observer. Although hidden messages can also be embedded in text transactions, their capacity is lesser compared to images capable of storing a large amount of data.

Finally, the block structure may differ depending on application and security requirements, which makes the presented solution flexible and ready-to-use in various areas. In addition, multiple image formats are supported, even within the same chain. The final configuration is up to the system architect, but it is good to start from the suggested initial setup (Table 1).

JPEG and other lossy formats cannot be used in some applications. Especially in medical imaging, when an image is used in diagnostics, information loss is unacceptable, and the image may only be compressed with the lossless algorithm. This concerns not only data transmission but also long- and short-term storage in the picture archiving and communication system (PACS). In such a scenario, imagechain is a very good choice with one limitation. When the image cannot be altered, only injection techniques are relevant, and substitution methods should not be used.

## 7. Discussion

The imagechains may be both public (open to everyone; users can store the copy of the chain, are able to add their own pictures and take part in the verification process) and private (participating in the network is based on a permission system). These approaches require a slightly different structure of blocks.

An example of a public imagechain is a collection of funny images distributed between all participants. The system may be designed to be anonymous or in a way that each user digitally signs his/her image, which additionally allows to earn reputation or unlock some achievements. It is also possible to create a consensus mechanism in which each participant decides if an image is good enough to be part of the chain and chooses whether to mine the block or to reject it. In this way, the verification can be seen as voting on pictures, and the majority of nodes play a trendsetter role. This is in opposition to the common solution of a centralized database of images stored on a server in which each user may vote + or − per picture. In such a case, a single user may create many fake accounts and achieve great impact with minimal effort. When the database is decentralized, voting is more absorbing because it consumes processing power. In this way, two goals are achieved: the content is more reliable, and trolling behavior is reduced. The users may also decide to only receive materials and do not contribute nor be involved in the decision process.

The important feature of the imagechain is that the chain elements are normal graphic files that can be viewed with standard software. Without the necessity to install additional external applications, the system is friendly to non-technical users and provides a high level of accessibility, which makes it suitable for use in public administration. One of the possibilities is to create an imagechain in which only authorized users are able to add new elements, but everyone may store a copy of the chain and participate in fraud detection. This solution not only allows verifying the digital signature and a hash but also preserves version history. It is an easy way to distribute digital images, e.g., maps to the public.

Another useful application is a family photo collection. It is an example of an imagechain with access limited only to family members. In this system, blocks may be designed to contain some additional information, for instance, when and where the photo was taken, a short description, etc. Every authorized user can add a new element to the album. The main advantage of using an imagechain to photo collection is that it automatically creates a distributed database that may be accessed by every family member. This type of storage helps to protect valuable photos that are impossible to recreate when missing.

Imagechain may also be applied in medical imaging to store treatment history. In such a case, a chain is not public as it contains the patient’s sensitive data. Instead, it may be accessed by the patient and medical personnel. The images are ordered chronologically, so the structure of the database depicts treatment progress with added timestamps. The patient is able to access and also verify the data, but only the doctor can add a new element. Various implementations are possible: one big chain for a single person or multiple chains for each treatment. Such a system can ease the sharing of medical data between hospitals, even if the patient is cared for in multiple institutions around the world. A number of countries sign international agreements providing mutual medical insurance for their citizens. Therefore, it is possible to create a network of medical institutions that share data. With a distributed database of images, when a new party joins the agreement, it is easy to grant access and provide faster treatment to the patient. It may also reduce the costs of accidental data loss in case of an accident (e.g., fire) because the data are automatically backed up by different institutions.

**Figure 5:**
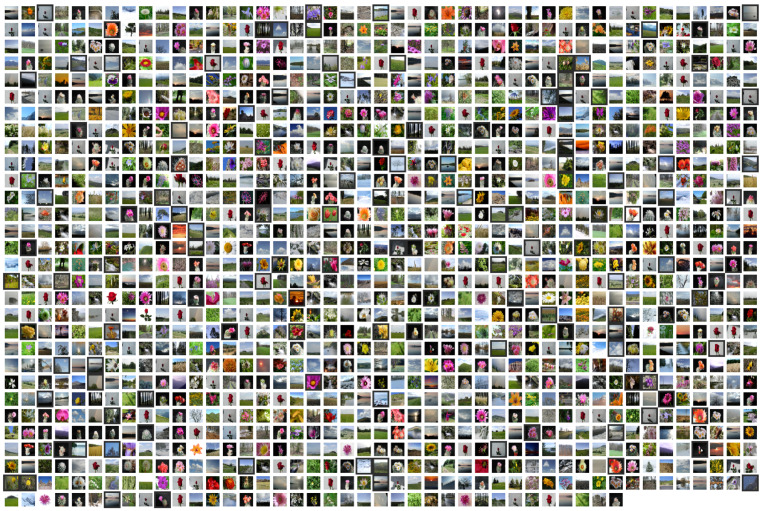
Image collection with hidden imagechain (pictures with dark borders).

The interesting property of imagechain is that it does not require any additional files, so it may be stored just as a collection of images. In other words, the whole chain may be saved in a single directory or filesystem. For external observers, such a directory is not different from any other directory containing random photos. Thanks to that feature, one may create hidden imagechains and use them in steganography [17]. In this case, a good choice of embedding method is an unusual algorithm that is hard to detect. The correct order of images is easy to determine by comparing their modification dates. Figure 5 is presented as an example of an image database, from which some are part of the imagechain. The selected images are marked with a dark border, which is not visible during normal usage. We chose the F5 algorithm to embed the blocks so that they are hard to detect. The used hash function is SHA256.

**Figure 6:**
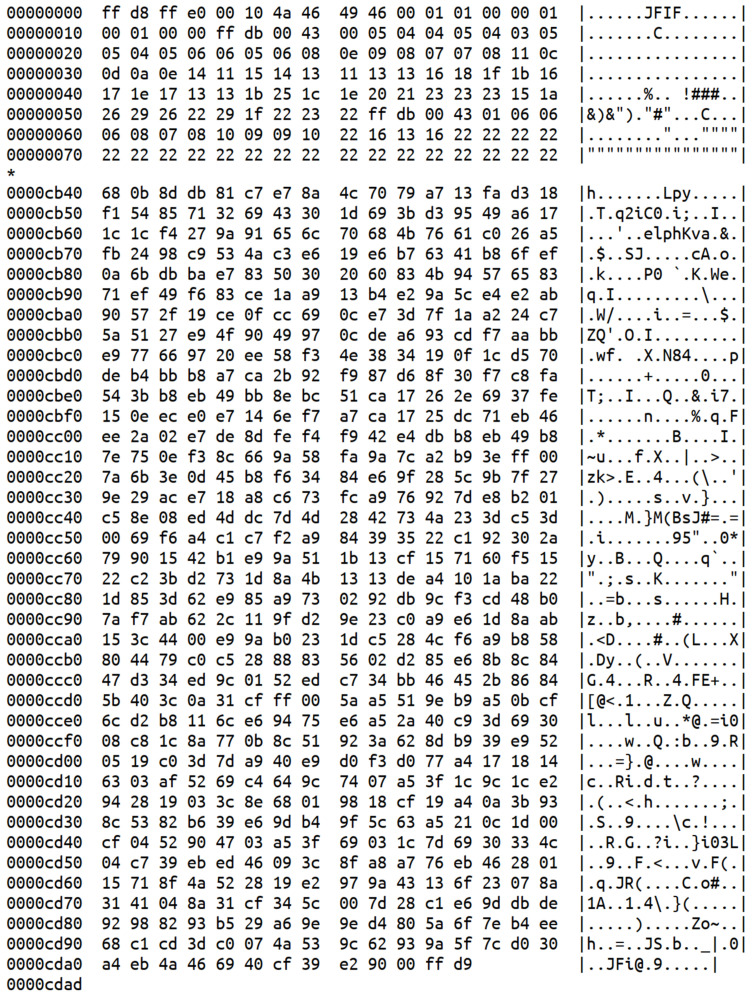
Hex dump of the image that contains a hidden block (begin and end).

Figure 6 presents the hex dump of one of the images from the imagechain. It may be compared to Figure 4, in which the chain is overt.

Imagechain may also be used in systems in which partial verification is required, but there is no space to store the whole blockchain. Such a situation is common in IoT devices or security camera settings when the new data replace the oldest entries. Storing the whole history from the beginning is not only impossible because of storage limitations [29], but usually also unnecessary, as a very small fraction of recorded material is interesting. The recordings are only used in case of accident, crime or other unusual situations. Their security is crucial because they may serve as evidence to prove someone guilty or innocent. The system should be able to detect tampering with data, for example, deletion or modification. A single image may be verified with a digital signature, but one cannot be sure that a picture has not been deleted. With imagechain, it is possible to verify the “snapshot” that consists of recordings from, for instance, one week. Imagechain links the images into a timeline structure that changes every time when the new data are added (at the same time, the oldest data are removed). However, the actual state of the chain may be analyzed for forgery or other suspicious actions. This is possible because the blocks are inseparable from pictures in the chain and cannot be silently removed. Additionally, the blocks may store metadata such as date and time or frame number instead of placing these data on the top of the frame; this enlarges the field of view significantly.

## 8. Conclusions

Presented in this article, imagechain is a new method of linking digital images to form a linear structure. The main advantage of the proposed solution is that data blocks are stored directly in images, thereby removing the necessity of a separated ledger. Intentional or accidental image modifications and other cases of data tampering are easy to detect with a hash function. This makes imagechain resistant to fraud in a similar way as the blockchain. Additionally, any image from the chain may be used separately as a normal file. However, even outside, it is still part of the chain and may be authenticated with respect to all previous pictures.

Imagechain supports various types of chains, including public and private. In addition, it leaves the room for various implementations that match individual requirements. It may be in simple or advanced form, with additional features adapted to needs. Therefore, we conclude that imagechain is a flexible solution that is suited for a variety of applications.

Future studies may cover various systems based on imagechain and also improvements of the framework itself. Another possible direction is to propose new data structures in which images are not linked sequentially but in a more complex way. They may find application in steganography and be adapted to the cloud environment.

## Figures and Tables

**Table 1:** Recommended setup for general use.

Embedding Method Class	Container Modification (Injection)
Carrier format	JPG or PNG
Embedding method	for JPEG—comment segment; for PNG—after IEND marker
Hash function	SHA256

## Data Availability

Data sharing not applicable.
